# Analytical Ultracentrifugation for Biopharmaceutical Characterization and Quality Control

**DOI:** 10.3390/ijms27136075

**Published:** 2026-07-07

**Authors:** Xiaojuan Yu, Wendan Chu, Qing Chang, Kaiyue Zhao, Zhaoxing Wang, Chengshi Zeng, Lan Wang, Chuanfei Yu, Wenqi Li

**Affiliations:** 1 State Key Laboratory of Drug Regulatory Science, NHC Key Laboratory of Research on Quality and Standardization of Biotech Products, NMPA Key Laboratory for Quality Research and Evaluation of Biological Products, National Institutes for Food and Drug Control, Beijing 102629, China; yuxiaojuan@nifdc.org.cn (X.Y.); 3325010579@stu.cpu.edu.cn (K.Z.); wanglan@nifdc.org.cn (L.W.); 2 Beijing Frontier Research Center for Biological Structure, Tsinghua University, Beijing 100084, China; chuwendan@mail.tsinghua.edu.cn (W.C.); changqing@mail.tsinghua.edu.cn (Q.C.); wangzhaoxing@mail.tsinghua.edu.cn (Z.W.); zengchengshi@mail.tsinghua.edu.cn (C.Z.); 3 Core Facility for Biomolecule Preparation and Characterization, Technology Center for Protein Sciences, Tsinghua University, Beijing 100084, China; 4 School of Pharmacy, China Pharmaceutical University, Nanjing 211198, China

**Keywords:** analytical ultracentrifugation, biopharmaceuticals, quality control

## Abstract

Modern biotechnology has rapidly developed, and biotechnological drugs have become the center of global drug research and development as an important aspect of clinical treatment. These drugs have unique advantages, such as strong species specificity and prominent targeting, and they are widely used in the treatment of various intractable diseases. However, their complex molecular structure, poor stability, and significant heterogeneity make quality control much more difficult than that of traditional small-molecule drugs that require high-precision analytical methods. Analytical ultracentrifugation (AUC) technology was pioneered by Theodor Svedberg during the early 20th century, and it has become an indispensable biophysical tool through technological innovation. This technology has unique advantages for the quality control of biotechnological drugs, including non-destructive detection, high resolution, and wide applicability. Therefore, AUC has been extensively adopted for the characterization of various biological products. This review systematically summarizes AUC technology applications in major categories of biotechnological drugs, with the aim to provide technical guidance and promote the standardized application of AUC.

## 1. Introduction

The rapid advancement of modern biotechnologies that include genetic engineering, cell engineering, protein engineering, and nucleic acid drug delivery technology has caused biotechnological drugs to evolve into vital components of global new drug research and development and clinical therapy. Biotechnology drugs are predominantly composed of biological macromolecules that are manufactured using living cells or biological systems. They exhibit unique merits such as high species specificity, prominent targeting properties, and definite therapeutic efficacy, and they cover recombinant proteins, antibody drugs, nucleic acid drugs, peptide drugs, vaccines, and cell and gene therapy products. Significant differences exist in the molecular structures, manufacturing processes, mechanisms of action, and quality attributes among the different biotechnological drug categories, collectively forming a diversified biopharmaceutical system [1].

Compared with traditional chemical drugs, biotechnological drugs are characterized by highly complex molecular structures and production processes dependent on living cell systems and loading conditions. Additionally, they can have inferior product stability and poor batch-to-batch consistency. Critical quality attributes such as aggregates, fragments, misassembled particles, empty capsids or partially packaged particles, heterogeneous nucleic acid encapsulation, and glycosylation heterogeneity may all compromise drug efficacy, safety, and immunogenicity. Therefore, as a key link in research and development and the production and marketing of biotechnological drugs, quality control (QC) is enormously challenging. The establishment of analytical methods that are capable of accurately characterizing these critical quality attributes serves as an essential foundation for biotechnological drug research and development (R&D), batch release, stability investigations, and process change evaluations. This issue has important theoretical and practical significance for the advancement and high-quality development of biopharmaceuticals, and to ensure clinical medication safety [2,3,4,5].

Analytical ultracentrifugation (AUC) is a classic biophysical characterization technology that originated from research on colloids and dispersed systems conducted by the Swedish chemist Theodor Svedberg during the early 20th century. He was subsequently awarded the Nobel Prize in Chemistry in 1926 for his related contributions. The first analytical ultracentrifuge developed by Svedberg laid the foundation for the determination of molecular weights and the sedimentation behavior research of biological macromolecules. Since then, AUC has undergone continuous upgrades due to instruments, optical detection systems, and data analysis software. The commercial Model E instrument to the modern Optima AUC platform, coupled with the improvements in analytical software that include SEDFIT, UltraScan, and BASIS, has allowed the resolution, quantitative capability, automation level, and compliant application performance of AUC to be remarkably enhanced. These advancements have made it an irreplaceable core tool in the biological macromolecule field [6,7,8,9]. In the biopharmaceutical sector, AUC has been widely applied in the research and development and QC of various biological products such as recombinant proteins, monoclonal antibodies (mAbs), vaccines, viral vectors, lipid nanoparticles (LNPs), and cell and gene therapy products [10,11,12,13,14,15].

AUC possesses unique advantages for the QC of biotechnological drugs. First, based on physical measurements of particle sedimentation behavior in a centrifugal field, AUC requires no stationary phase or column packing. This aspect allows for non-destructive analysis in native or near-original formulation solution environments to truly reflect the native conformation and intermolecular interaction of biological macromolecules [16,17]. Second, AUC effectively characterizes the size, shape, molecular weight distribution, and aggregation state of biological macromolecules. It also delivers high resolution for the distribution characterization of heterogeneous samples with variable particle loading rates and empty capsid ratios [18,19,20]. Third, integrated with multiple detection modes including ultraviolet, interference, and fluorescence detection, AUC is applicable for the analysis of small-molecule peptides, protein complexes, and nanoparticle systems such as adeno-associated virus (AAVs), LNPs, and extracellular vesicles (EVs). Additionally, it supports data processing under good manufacturing process (GMP) environments via compliant software and standardized workflows, providing reliable technical support for the quality and safety of biotechnological drugs [21,22,23].

Although several reviews have been published on the AUC methodology and its partial biopharmaceutical applications, evident limitations restrict their ability to meet current QC demands for diversified complex biotechnological drugs. First, most prior reviews have only focused on single modalities such as mAbs or recombinant AAVs, thus failing to integrate popular clinically emerging products, including mRNA-LNP vaccines, oncolytic viruses, glucagon-like peptide-1 (GLP-1) peptides, EVs, and glycoprotein conjugates, resulting in fragmented industry-wide application guidance. Second, most previous literature has emphasized fundamental sedimentation biophysics, with a weak correlation between AUC readouts and regulatory-mandated critical quality attributes, as well as a lack of industrial registration and batch release cases tailored to pharmaceutical labs.

To address the above research gaps, this review proposes original analytical perspectives. First, a full-spectrum classification framework spanning mainstream biopharmaceutical platforms is established, which systematically integrates AUC application scenarios for antibody drugs, viral gene therapy vectors, multi-platform vaccines, peptide therapeutics, EVs, recombinant proteins and glycoprotein products. This framework provides a one-stop reference for laboratories with diversified biotech pipelines. Second, the orthogonal evidentiary value of AUC in biosimilar comparability assessment, regulatory testing of gene therapy vectors, vaccine formulation optimization, and long-term stability profiling is systematically elaborated upon. This effort consolidates fragmented state-of-the-art technical datasets into compliant, readily implementable QC workflows, thereby advancing the standardized, scalable, and regulatory-aligned deployment of AUC across the life cycle of biotherapeutic products.

## 2. AUC Applications in Antibody Drugs

**Figure 1:**
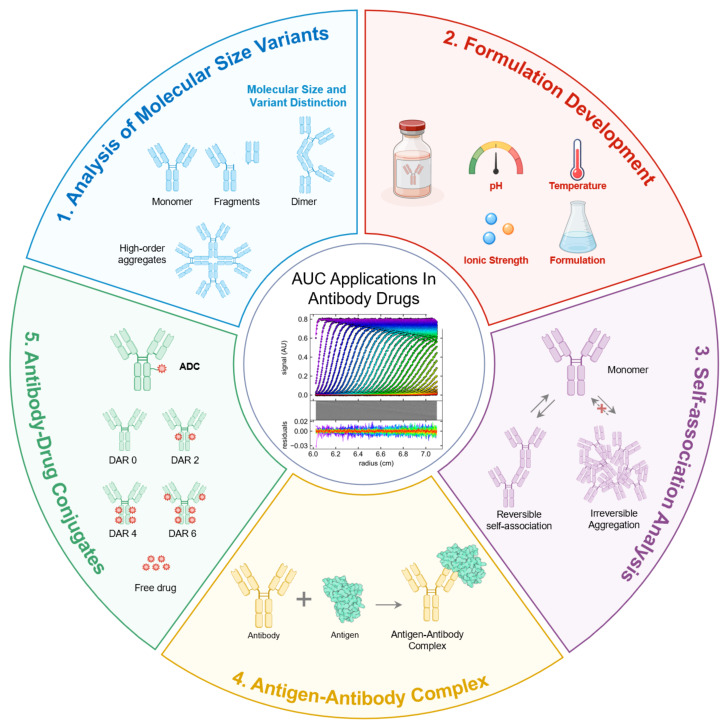
Analytical ultracentrifugation applications in antibody drugs.

AUC is a powerful biophysical technique widely applied across critical stages of antibody drug development. It facilitates the analysis of molecular size variants, formulation optimization, self-association behavior, antigen–antibody complex formation, and antibody–drug conjugate (ADC) characterization. As illustrated in Figure 1, AUC offers key quantitative and qualitative insights for antibody QC, including biosimilar similarity assessment, formulation stability evaluation, and the characterization of complex biotherapeutic interactions and conjugates.

### 2.1. Analysis of Molecular Size Variants in Antibody Drugs

Monoclonal antibody drugs require strict monitoring of their molecular size variants that include monomers, fragments, dimers, and higher-order aggregates. Sedimentation velocity AUC (SV-AUC) can separate and quantitatively integrate different components through sedimentation coefficient distribution profiles, and this technique is commonly adopted as an orthogonal verification tool for size exclusion chromatography (SEC), size exclusion chromatography combined with multiangle light scattering (SEC-MALS), capillary electrophoresis-sodium dodecyl sulfate (CE-SDS), and other analytical techniques. The Federal Drug Administration (FDA) guideline for comparative analytical assessments of therapeutic protein biosimilars emphasizes that adequate comparative analyses should be conducted that focus on quality attributes related to product quality, safety, and efficacy, among which molecular size variants and aggregates are core evaluation indicators. In practical research, AUC is frequently used as an orthogonal analytical method to compare such quality attributes.

ABP 501 is an adalimumab biosimilar developed by Amgen that will be used as an example. Multiple orthogonal methods have been adopted to systematically compare the quality similarity between ABP 501 and reference formulations that are marketed in the United States and the European Union. Real-time monitoring of the sample sedimentation processes via SV-AUC was used to accurately quantify the distribution proportions of monomers and different aggregates. The results confirmed a high consistency in the overall aggregation level between ABP 501 and the two reference formulations [24]. In a similarity evaluation of trastuzumab biosimilar HLX02, SV-AUC was also incorporated into the orthogonal analytical system to compare the differences in high-molecular-weight components, fragments, and aggregation states between HLX02 and Herceptin sourced from the EU and domestic markets to verify the high similarity of their relevant quality attributes [25]. Specifically, CE-SDS is an indispensable orthogonal technique for analytical similarity assessments of biosimilar products, as demonstrated in the aforementioned case studies. Consistent with regulatory guidance from the FDA, European medicines agency (EMA), and National medical products administration (NMPA), CE-SDS is uniquely capable of characterizing covalent size variants of therapeutic antibodies. Nevertheless, SV-AUC exhibits distinct technical advantages relative to CE-SDS. Notably, measurements are performed directly in the original native formulation without denaturing agents, thus enabling authentic profiling of the intrinsic aggregation behavior of biotherapeutics under physiologically relevant buffer conditions.

In addition, Bou-Assaf et al. summarized the practical key points of SV-AUC for the aggregate quantification of therapeutic antibodies. They highlighted the importance of experimental design, concentration selection, optical detection, data fitting, and a statistical evaluation to obtain reliable quantitative results [26].

### 2.2. Formulation Development of Antibody Drugs

During antibody formulation development, the pH, ionic strength, protein concentration, excipient composition, storage temperature, and storage duration may all affect the conformational stability, self-association, and aggregation tendency of antibodies. AUC screens the formulation conditions that maintain protein conformational stability by detecting differences in antibody sedimentation behaviors under various formulation conditions. This provides scientific evidence for drug formulation design and stability evaluation.

In a study performed by AstraZeneca, SV-AUC was used to evaluate the intermolecular interactions of two monoclonal antibodies (mAb-A and mAb-B) under co-formulation conditions. They analyzed the sedimentation coefficient distribution ls-g∗(s) profiles, and the absence of heterologous aggregates during the co-formulation was directly verified. The sedimentation profile of the co-formulation was highly consistent with that of individual monomers, and no intermolecular interactions were observed when adjusting the co-formulation concentration (0.2–2.0 mg/mL) and antibody ratio (4:1 to 1:1). Combined with orthogonal verification via isothermal titration calorimetry (ITC), dynamic light scattering (DLS), and other technologies, a comprehensive interaction evaluation system was then constructed that demonstrated the reliability of AUC under complex practical formulation environments [27].

### 2.3. Self-Association Analysis of Antibody Drugs

Antibody self-association includes reversible self-association and irreversible aggregation. In a collaborative study, Mehta et al. used SV-AUC to monitor the aggregation behavior of monoclonal antibodies incubated at 37 °C for different durations in guanidine hydrochloride (GdnHCl) solutions with concentrations that ranged from 0 to 2.0 M. The results showed that mAbs remained in a monomeric state (s_20,w_ ≈ 7.3 S) without aggregation at low GdnHCl concentrations. Partial unfolding occurred at moderate GdnHCl concentrations, and this was accompanied by the formation of soluble aggregates over time (accounting for up to 20%). Further unfolding was observed at high GdnHCl concentrations, 1.8 M exhibited slight aggregation, while 2.0 M showed no obvious aggregation. This result indicated that aggregation was associated with the partially unfolded state. Combined with differential scanning calorimetry (DSC) data, researchers concluded that the unfolding of the CH2 domain was the key driver of aggregation. The DSC results revealed that the thermal transition peak of the CH2 domain disappeared at GdnHCl concentrations ≥ 1.2 M. This indicates the unfolding of the domain in solution, while AUC simultaneously detected aggregate formation. This finding clarified that partial unfolding is a prerequisite for aggregation and highlighted the high-resolution advantage of AUC for capturing dynamic conformational changes [28].

Researchers from an AstraZeneca subsidiary systematically investigated the reversible self- association behavior of antibody mAbA using a multi-technique combination. The SV-AUC results showed that the peak of the sedimentation coefficient distribution significantly shifted from 7.5 S to 11.5 S as the protein concentration increased from 0.25 mg/mL to 9.8 mg/mL. This result suggested the existence of concentration-dependent higher-order associates in the system. To further quantitatively characterize the reversible self-association process, a global fitting of the data under multiple concentration gradients and rotational speeds was performed via sedimentation equilibrium analytical ultracentrifugation (SE-AUC). This step successfully established a monomer-trimer-hexamer reversible self- association model [29]. The results of these studies indicate that AUC can not only quantify aggregate proportions but also distinguish rapidly equilibrated reversible self-association from long-term irreversible aggregation. This differentiation is critical to comprehensively evaluate the solution behavior characteristics of antibodies, and it is particularly valuable for the development of high-concentration antibody formulations. However, certain limitations of AUC should be recognized, including the requirement for expert data analysis, model-dependent data interpretation, and nonideal solution behaviors at high sample concentrations.

### 2.4. Detection of Antigen–Antibody Complexes

In the characterization of antibody–antigen complexes, AUC can be used to identify complex formations, determine stoichiometric ratios, and characterize distribution heterogeneity. Arthur et al. conducted a systematic study on the complex formation process between denosumab and RANKL using SV-AUC. By accurately determining the sedimentation coefficient distribution, multiple complex components with different stoichiometric ratios were identified. Among them, the 3D2R complex (three denosumab molecules bound to two RANKL trimers) was identified as the dominant component, with a sedimentation coefficient of approximately 14 S at 20 °C and 20 S at 37 °C, exhibiting significant temperature dependence. A series of intermediate complexes that included 1D2R (sedimentation coefficient ≈ 11.5 S) and 3D1R (sedimentation coefficient ≈ 12.5 S) were also detected. These intermediate species appeared at the initial reaction stage or at a low temperature. They gradually converted to the more stable 3D2R complex over time and with increasing temperature. A systematic analysis of the c(s) distribution profiles under different molar ratios (3:1, 1:1, 1:2, and 1:3) fully demonstrated the excellent resolution capability of AUC in complex multicomponent systems. Notably, denosumab binds to the receptor activator of nuclear factor Kappa-B ligand (RANKL) in a unique 3:2 stoichiometric pattern that complements the traditional monovalent antigen–antibody binding model. The 3D2R complex remains dominant even at a low concentration of 6 μg/mL, a characteristic highly consistent with in vivo pharmacokinetic profiles. This provides an important theoretical basis for the optimization of clinical administration regimens [30].

AUC provides powerful technical support for the analysis of complex associates during the detection of bispecific antibody complexes. In a study of the interaction between MEDI4276 and HER2, Oganesyan et al. adopted multi-signal sedimentation velocity analytical ultracentrifugation (MSSV-AUC) to simultaneously collect UV280-nm, UV250-nm, and interference signals. They accurately calculated the molar ratio of each component in the complex using the specific absorbance increment of each protein. The results showed that MEDI4276 formed multiple complexes with HER2 at ratios of 2:1, 3:2, and 5:3, with sedimentation coefficients ranging from 10 S to 32 S and corresponding molecular weights spanning 364 kDa to 2.1 MDa. Compared with conventional monoclonal antibodies, the complex exhibited significantly higher heterogeneity. This result confirmed the complex binding behavior induced by the bispecific design [31].

Similarly, in a study of the interaction between the bispecific antibody BiS3Ab and IL-6, BiS3Ab formed complexes with IL-6 that covered a wide sedimentation coefficient range from 0 S to 80 S, which corresponded to molecular weights of 200 kDa to 3 MDa. Approximately 70% of the complexes were large aggregates, 29% were medium-sized complexes, and only 1.4% were small complexes. Compared with parental monoclonal antibodies (mAb1 and mAb2), the complexes showed high heterogeneity, reflecting the complexity of the interaction between bispecific antibodies and target ligands [32].

Compared with traditional chromatographic methods, AUC exhibits prominent advantages in the characterization of antibody–antigen complexes. Based on a first-principle analysis via the Lamm equation, AUC requires no stationary phase. This effectively avoids complex damage caused by column adsorption and shear force. Experiments are conducted under native buffer conditions to maximally preserve the natural protein conformation and the authenticity of intermolecular interactions. By synchronously acquiring multiple UV wavelengths and interference light signals and utilizing the specific absorbance increment of each component, AUC enables precise quantitative determination of complex compositions. This multi-signal detection strategy improves the accuracy and reliability of quantification [33,34].

### 2.5. Analysis of Antibody–Drug Conjugates

The rapid development of biopharmaceutical technology has allowed for the continuous emergence of complex conjugated drugs such as antibody–drug conjugates (ADCs), radioimmunoconjugates (RDCs), antibody–oligonucleotide conjugates (AOCs), antibody–peptide conjugates (APCs), peptide–drug conjugates (PDCs), and peptide–nanoparticle conjugates (PNCs). Diverse conjugation modes and high structural heterogeneity impose higher requirements on their QC. Taking ADCs as an example, the conjugated small-molecule toxins are strongly hydrophobic, and an increase in the drug-to-antibody ratio (DAR) significantly enhances intermolecular hydrophobic interactions, thereby triggering aggregation. In addition, intermolecular electrostatic interactions of conjugated drugs also serve as an important factor that drives aggregate formation. These characteristics necessitate the precise monitoring of aggregate content throughout the drug development lifecycle. For structurally complex and heterogeneously distributed conjugated systems, AUC acts as a critical orthogonal tool alongside SEC, multi-angle light scattering (MALS), mass spectrometry, and electrophoresis for the characterization of aggregates, conjugate distribution, and complex composition.

Falvo et al. systematically analyzed ferritin-based cisplatin-encapsulated nanoparticles (HFt-Pt) and their antibody Ep1 conjugates (HFt-Pt-Ep1) using AUC. The sedimentation coefficients and molecular weights of the HFt-Pt, Ep1 antibody, and HFt-Pt-Ep1 conjugates were successfully determined. The results showed that HFt-Pt had a sedimentation coefficient of 15.1 S that corresponded to a molecular weight of approximately 500 kDa. The Ep1 antibody exhibited a sedimentation coefficient of 6.8 S, with a molecular weight of approximately 145 kDa; and the HFt-Pt-Ep1 conjugate had a sedimentation coefficient of approximately 25.6 S and a molecular weight of approximately 900 kDa. These data confirmed the successful construction of the conjugate and revealed that each HFt-Pt nanoparticle was linked to three Ep1 antibody molecules with a stoichiometric ratio of 1:3. The AUC results effectively verified the uniformity and stability of the conjugate [35]. Furthermore, MSSV-AUC can be used to determine the drug-to-antibody ratio (DAR) of ADCs. Clardy et al. measured antibody–drug conjugates with different degrees of heterogeneity and verified the results via orthogonal methods that included high-performance liquid chromatography (HPLC), matrix-assisted laser desorption/ionization (MALDI), and SEC-MALS, proving that MSSV-AUC can provide accurate information on the critical quality attributes of ADC samples [36].

Additionally, artifacts from hydrophobic interactions are frequently observed in the SV-AUC characterization of ADCs and hydrophobic mAbs. Several standardized operational workflows are summarized to reduce such measurement biases. First, rigorous thermal regulation and adequate equilibration must be performed. SV-AUC measurements are routinely conducted at 20 °C, with temperature fluctuations restricted within ±0.5 °C across the entire analytical run. Moreover, after sample loading, the rotor requires a 1–2 h equilibration period under vacuum conditions to remove thermal gradients and suppress the generation of transient hydrophobic oligomers emanating from incomplete thermal stabilization. Second, appropriate sample loading absorbance at 280 nm should be maintained above 0.5 OD. Concentrations falling below this threshold yield poor signal-to-noise ratios for trace oligomeric species, making them indistinguishable from baseline noise and introducing bias into aggregate quantitation. Third, accurate centerpiece alignment is indispensable. Optical alignment is the gold standard, although mechanical alignment can be adopted as a viable substitute. A brief, low-speed centrifugation step before alignment is recommended to evacuate entrapped air bubbles in centerpieces, thus eliminating spurious high-molecular-weight peaks caused by convective disturbances.

## 3. AUC Applications in Viral Vector Products

### 3.1. Analysis of Adeno-Associated Virus

**Figure 2:**
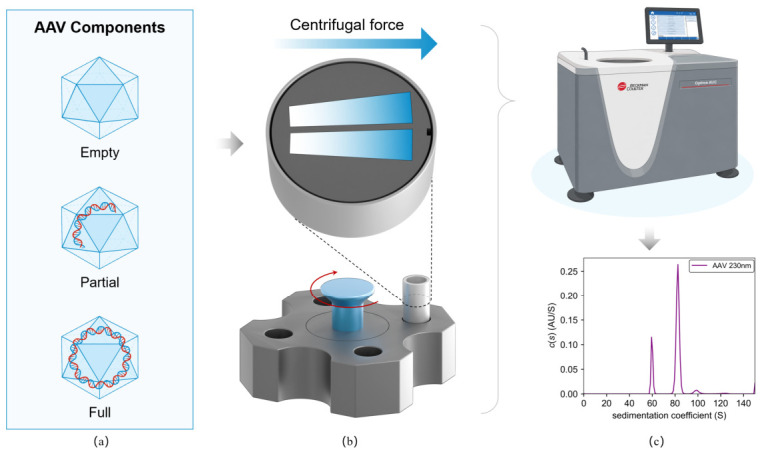
Workflow of sedimentation velocity analytical ultracentrifugation for adeno-associated virus quality control. (**a**) Adeno-associated virus samples contain heterogeneous populations including empty, partially filled, and full capsids with different sedimentation coefficients. (**b**) Under centrifugal force in an analytical ultracentrifugation cell, these capsid populations are separated based on their size, shape, and density. (**c**) UV detection is employed to generate sedimentation coefficient distribution profiles, which resolve distinct peaks corresponding to empty, partial, and full capsids, enabling quantitative assessment of packaging efficiency and sample heterogeneity.

Recombinant adeno-associated virus (rAAV) has become one of the most widely used viral vectors in gene therapy. To date, multiple AAV gene therapy drugs have been approved by the FDA and EMA, and hundreds of clinical trials are ongoing worldwide [37,38]. Accurate characterization of critical quality attributes, such as the empty capsid ratio and packaging integrity of rAAV, is essential to ensure the safety and efficacy of gene therapy products. Compared with mass photometry (MP), charge detection mass spectrometry (CDMS), and SEC-MALS, AUC has demonstrated superior performance in empty capsid ratio resolution and genomic length resolution (Figure 2) [39,40,41,42,43].

A research team from the National Institutes for Food and Drug Control of China systematically validated the specificity, precision, accuracy, and linearity of the SV-AUC method for quantifying the proportions of empty capsids, partially packaged particles, and full capsids of AAV. This established a standard SV-AUC protocol for the detection of AAV empty/full capsid ratios and provided a basis for the standardized application of AUC in the QC of AAV gene therapy drugs [44]. A high-speed SV-AUC (hs-SV-AUC) strategy was proposed to improve the resolution. Larson et al. adopted a high rotational speed of 45,000 rpm and low temperature conditions (5–10 °C). This protocol significantly increased the separation distance of particles with different sedimentation coefficients, while low temperatures reduced the sample diffusion coefficient to further improve the resolution [45]. Kokona et al. systematically evaluated the resolution limit of hs-SV-AUC through combined experiments and simulations. Two purified AAV variants with a genomic length difference of 696 nucleotides (NT) were mixed at a 1:1 ratio and analyzed using hs-SV-AUC at 45,000 rpm and 5 °C. The results demonstrated that hs-SV-AUC achieved the baseline separation of the two particles, while a conventional low-speed SV-AUC (15,000 rpm) failed to distinguish them completely. Further simulation studies indicated that hs-SV-AUC could resolve AAV particles with a genomic length difference of approximately 300 NT, corresponding to a sedimentation coefficient difference of 1–1.5 S at 5 °C. Such resolution is significantly superior to that of mass photometry and conventional low-speed AUC protocols [46].

The quantitative detection of AAV by AUC is also affected by experimental parameters and optical properties. Luo et al. investigated the influence of cell alignment deviation on the quantitative results of AAV9 samples using an optical alignment system. They found that when the cell deflection angle increased from 0° to +2° (clockwise), the apparent content of the high-sedimentation (HS) components rose sharply from approximately 3.6% to nearly 30% at 20,000 rpm. In contrast, the HS component content only increased from 3.6% to 13.0% at a deflection of −2° (counterclockwise) [47]. Yamaguchi et al. determined the extinction coefficients of empty and full capsids of AAV serotypes 2, 5, 6, 8, and 9 at wavelengths of 230, 260, and 280 nm using SV-AUC combined with interference and multi-wavelength detection. The results showed that the extinction coefficients of full capsids at 260 nm and 280 nm were significantly higher than those of the empty capsids. Hence, the simple adoption of identical extinction coefficients for calculation may lead to the quantitative deviation of empty/full capsid ratios, especially under low wavelength conditions or with variable genomic packaging lengths [48]. These studies indicated that the application of AUC for AAV QC requires attention not only to data fitting but also to methodological details that include cell assembly, detection wavelengths, extinction coefficient calibration, and system suitability reference materials.

### 3.2. Analysis of Lentiviral Vectors

Lentiviral vectors (LVVs) are retrovirus-derived gene delivery systems capable of integrating their genome into the host cell DNA to achieve long-term stable expression of exogenous genes. Modified from human immunodeficiency virus (HIV) and other retroviruses, LVVs retain the ability to infect non-dividing cells while removing pathogenic genetic elements via genetic engineering. This improves their safety for clinical application.

SV-AUC exhibits remarkable advantages for the QC of LVVs. For LVV samples with low impurity contents, distinct peaks with a sedimentation coefficient of approximately 650 S can be observed by AUC detection, and this is highly consistent with the characteristic sedimentation coefficient (~600 S) of retroviruses. This result indicates that SV-AUC can specifically identify LVV particles. For high-impurity samples, a two-step centrifugation strategy is adopted to improve the separation efficiency. First, a preliminary separation of the LVV particles from large particulate impurities is performed at 7000 rpm. Subsequently, the rotational speed is increased to 42,000 rpm to further resolve small-molecule impurities such as human albumin and other protein components. This method enables the simultaneous acquisition of sedimentation profiles of LVVs and coexisting impurities during a single experiment. A distinct peak near 650 S was observed in the SV-AUC results, with an A_260_/A_280_ ratio of approximately 1.3. This result was consistent with the spectral characteristics of nucleic acid- and protein-composed LVV particles, further verifying the reliability of this method [49].

### 3.3. Analysis of Oncolytic Viruses

Oncolytic viruses (OVs) are viral preparations that selectively infect and replicate in tumor cells. They ultimately lyse tumor cells and activate the body’s anti-tumor immune response. They have promising application prospects in tumor immunotherapy. Multiple OV products based on different viral backbones have been clinically approved, including T-VEC (Imlygic) derived from herpesvirus, H101 (Ankerui) derived from adenovirus, and Rigvir derived from echovirus. The QC of OVs faces unique challenges. The empty capsid viruses, aggregates, and infectious intact viral particles have subtle differences in hydrodynamic properties such as particle size, molecular weight, and surface charge, making it difficult for single conventional analytical methods to fully characterize their quality attributes. SV-AUC enables efficient separation and quantitative determination of different viral particles in near-physiological solution environments. For example, for herpesvirus-derived OVs, SV-AUC can clearly distinguish empty capsid particles (~713 S), intermediate particles (~924 S), and intact viral particles (~1205 S) [50]. For enterovirus EV71, SV-AUC effectively differentiates two assembly states that include 80 S empty particles and 150 S intact particles. Additionally, the sedimentation coefficient and relative percentage content of each viral component can be obtained using a single SV-AUC run [51].

However, when applying AUC for viral analysis, attention should be paid to avoiding artificial aggregation of large viral particles via three key approaches. First, the rotor speed should be restricted to ≤12,000 rpm (7000 rpm recommended for LVV) to avoid shear-induced envelope rupture and particle collision [52]. Second, simultaneous absorbance detection should be implemented at 260 nm and 280 nm to distinguish impurities, intact virions, and viral aggregates based on the A_260_/A_280_ absorbance ratio. Third, AUC should be complemented by orthogonal techniques such as TEM, NTA, and an infectious experiment, as AUC cannot generate direct readouts of sample function or infectivity.

## 4. AUC Applications in Vaccines

**Figure 3:**
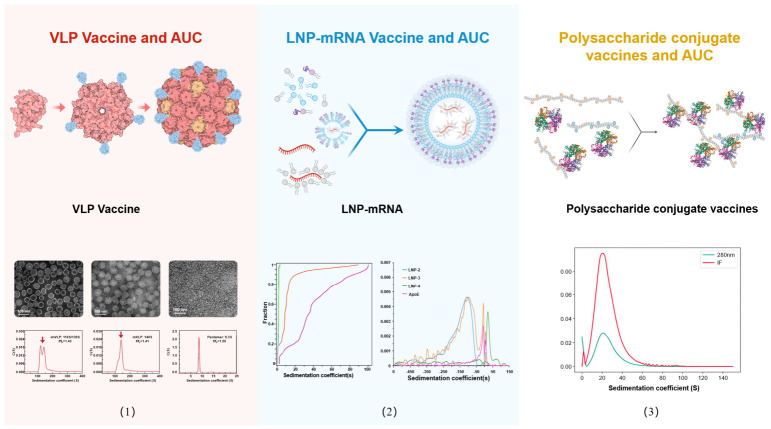
Analytical ultracentrifugation (AUC) applications in three major vaccine platforms. (**1**) For virus-like particle (VLP) vaccines, AUC evaluates assembly states (pentamers, immature/mature VLPs) and structural homogeneity, complementing TEM [53]; (**2**) For lipid nanoparticle (LNP-mRNA) vaccines, it resolves loaded/unloaded LNP subpopulations and polydispersity, thus supporting formulation and stability assessment [54,55]; (**3**) For polysaccharide conjugate vaccines, dual-signal AUC quantifies compositional heterogeneity and polysaccharide/protein ratios [56]. Figure 3 (**1**) adapted from [53] under the CC BY 4.0 license; Figure 3 (**2**) adapted from [53,54] under the CC BY 4.0 license; Figure 3 (**3**) adapted from [55] under the CC BY 4.0 license.

AUC is a versatile tool for characterizing diverse vaccine platforms, including virus-like particle (VLP), LNP-mRNA, and polysaccharide conjugate vaccines. It enables the analysis of assembly integrity, particle heterogeneity, and compositional distribution, thus addressing key challenges in vaccine QC and formulation development. Figure 3 shows AUC applications across these three major vaccine modalities.

### 4.1. Analysis of VLP Vaccines

Virus-like particles (VLPs) are nanoscale particles self-assembled from viral capsid proteins. They feature highly ordered structures and repetitive antigenic epitopes that mimic the spatial conformation of native viruses without containing viral genetic materials. As an emerging vaccine platform, VLP vaccines show prominent advantages in preventive medicine and they have been successfully applied in the R&D and industrialization of vaccines against human papillomavirus (HPV), hepatitis B virus (HBV), hepatitis E virus (HEV), and other pathogens. During VLP vaccine development, the assembly integrity, structural stability, particle homogeneity, and purity directly determine the vaccine immunogenicity and safety. Analytical ultracentrifugation enables real-time and dynamic monitoring of VLP particles under near-physiological solution environments. This allows for reliable data support for structural integrity evaluation, process optimization, and stability research [57,58].

In a study of a trivalent chimeric vaccine targeting HPV18/45/59, Chi et al. applied AUC to detect the sedimentation coefficients of chimeric VLPs and wild-type particles. Along with the results from transmission electron microscopy, high-performance size-exclusion chromatography and differential scanning calorimetry, it was verified that the assembled particles shared highly similar particle size, morphology and thermal stability with wild-type counterparts, proving the correct assembly of VLPs [59]. In structural research of the HPV59 L1 protein, AUC accurately distinguished L1 pentamers, immature VLPs, and mature VLPs. This result directly reflected the improvement of assembly homogeneity by the in vitro VLP maturation process through sedimentation coefficient differences [53].

In research on virus-like particles and viral structures, AUC has also been used to analyze the interaction between viral proteins and nucleic acids. For instance, Zhao et al. adopted AUC and other methods to study the nucleic acid-induced dimerization process of the HIV-1 Gag protein, clearly distinguishing Gag/NA complexes at stoichiometric ratios of 1:1 and 2:1 and clarifying the assembly mechanism of NA-induced Gag protein dimerization. This result provides an important basis for the assembly regulation of HIV-1 VLPs [60]. The results of these studies demonstrate the significant value of AUC for investigating viral particles and their assembly intermediates, while specific analytical protocols need to be matched with the virus type, particle composition, and research objectives.

### 4.2. QC of mRNA Vaccines

Lipid nanoparticles serve as the dominant delivery vectors for mRNA vaccines and nucleic acid drugs. Their particle size distribution, RNA loading efficiency, empty particle proportion, and internal structural homogeneity directly determine their delivery efficiency and safety. Conventional techniques, such as DLS and cryo-transmission electron microscopy (cryo-TEM), are unable to distinguish between unloaded and drug-loaded subpopulations, quantify polydispersity, or reflect the true solution state. AUC shows high value in addressing the limitations of conventional methods to resolve LNP polydispersity, heterogeneous drug loading, and insufficient resolution.

SV-AUC is capable of synchronously sensing particle size, shape, and density. It can also distinguish sedimenting (mRNA-loaded) and floating (unloaded/low-loaded) LNP subpopulations in a single experiment. The nucleic acid distribution is directly tracked via 260 nm UV signals that reveal the multi-peak distribution and subpopulation heterogeneity that cannot be identified by traditional methods. Thaller et al. proposed that SV-AUC could be used as a stability-indicating method for mRNA-LNPs to monitor changes in the LNP sedimentation behavior and heterogeneity under different treatment conditions. Zhao et al. further developed a flotation coefficient distribution analysis method for LNPs, proving that SV-AUC can resolve the high-resolution floating distribution profiles of lipid nanoparticles [61,62].

In LNP formulation characterization, AUC can accurately resolve the effects of different ionizable lipids and preparation processes on particle structure. Studies have confirmed that LNPs prepared by microfluidics exhibit smaller particle size and lower polydispersity than those produced by bulk mixing, and AUC can quantitatively distinguish significant differences in their sedimentation coefficient distribution and correlate with drug loading efficiency [63].

### 4.3. QC of Conjugate Vaccines

Polysaccharide conjugate vaccines are novel vaccines that are formed by covalently linking bacterial capsular polysaccharides to protein carrier molecules. They can effectively induce T-cell-dependent immune responses and overcome the limitations of traditional polysaccharide vaccines, such as weak immunogenicity and inability to induce long-term immune memory.

Researchers have determined the polysaccharide proportion in polysaccharide conjugate vaccines utilizing the dual-signal detection function of AUC. Two polysaccharide conjugate vaccines with tetanus toxoid as the carrier protein and significant differences in the protein content were selected as research objects. The UV absorbance at 280 nm and interference signals were collected simultaneously. A broad sedimentation coefficient distribution was observed for both vaccines, and this was consistent with the wide molecular weight distribution of polysaccharide conjugate vaccines detected using MALS technology in previous studies. The result reflected significant compositional heterogeneity of the samples in solution. Based on the difference between the absorbance and interference dual signals, the polysaccharide proportion in vaccines was calculated using AUCAgent software (v1.8.8). Three replicate tests were performed for each vaccine, and they all showed good consistency among replicate samples. This method serves as an orthogonal verification tool for component analysis with other analytical technologies [56].

In addition to the above-mentioned vaccines, AUC is also widely applied for the analysis of subunit vaccines, toxoid vaccines, and other vaccine platforms. Such vaccines are based on specific antigen units that include proteins, polysaccharides, toxoids, and their physicochemical properties, such as the aggregation state, conformational homogeneity, and stability that directly affect immunogenicity and safety. AUC can provide key data support for R&D, batch release, and stability evaluation of vaccines [64,65].

## 5. AUC Applications in Peptide Drugs

**Figure 4:**
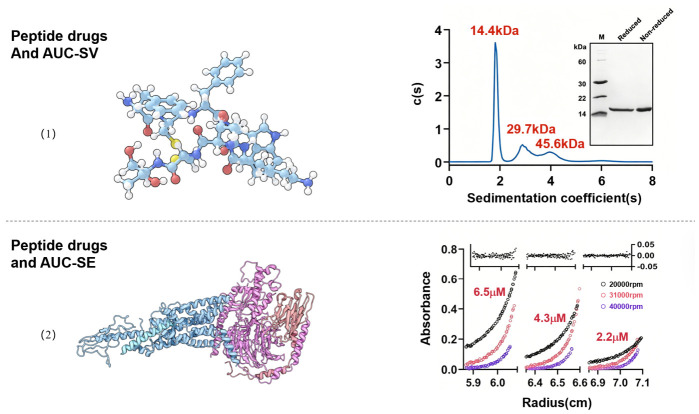
Analytical ultracentrifugation application in peptide drugs. (**1**) AUC-SV for peptide drug aggregation analysis. The c(s) distribution profile shows sedimentation coefficient peaks corresponding to monomeric and oligomeric peptide species, with molecular weights estimated at 14.4, 29.7, and 45.6 kDa, complemented by SDS-PAGE validation. (**2**) AUC-SE for self-association studies. The absorbance profiles at multiple rotor speeds and concentrations enable global fitting to ascertain that the binding affinities were 4.5 and 26.9 μM for the monomer-dimer and monomer-trimer [66]. Figure 4 was adapted from [65] under the CC BY 4.0 license.

Peptide drugs are biological agents that are intermediate between small-molecule chemical drugs and macromolecular protein drugs. With high biological activity, strong targeting specificity, and relatively favorable safety profiles, they play an important role in the prevention and treatment of tumors, metabolic diseases, infectious diseases, and autoimmune diseases. An aggregation state analysis is a core link in evaluating the stability, safety, and efficacy of peptide drugs during their R&D and QC. AUC provides accurate and reliable experimental data support for the multi-dimensional research of peptide drugs via two core modes: SV-AUC and SE-AUC (Figure 4).

GLP-class peptides are important drugs for the treatment of type 2 diabetes and obesity. Their solution aggregation state, conformational changes, and stability directly affect pharmacological efficacy, formulation development, and medication safety. One of the core applications of AUC is to accurately characterize the associated state of GLP peptides and clarify their assembly patterns under different concentrations and pH conditions. In a study of teduglutide, researchers used SV-AUC to obtain the sedimentation coefficient distribution via a c(s) analysis within the concentration range of 0.135–2.17 mg/mL. The results showed that the peptide exhibited reversible aggregation characteristics with increasing concentration. Combined with the global fitting of SE-AUC data, the assembly pathway was finally determined, and the relevant aggregation constants and sedimentation coefficients of each aggregated state were calculated. In addition, based on the obtained sedimentation coefficient and friction ratio (f/f0), it was inferred that monomers adopt a moderately compact conformation, while pentamers exhibit a slightly extended conformation. This provides indirect evidence for the structural characterization of the aggregate [67].

Studies have found that GLP-1 forms low-molecular-weight oligomers, and such oligomers are mostly off-pathway to fibrillation that cannot be directly converted into amyloid fibrils, whose stability is critical for the long-term storage of drug formulations. An SV-AUC analysis of freshly prepared GLP-1 samples and samples incubated for 7 days detected dimers and higher-order oligomers. This result clarified the temporal variation pattern of the oligomer distribution. Combined with SEC data, it was confirmed that AUC can detect transient unstable oligomers that cannot be captured by SEC, making up for the limitations of a single analytical technology [68].

In the research on the interaction between the GLP-1 receptor (GLP-1R) and peptide ligands, AUC provides important support for the oligomerization characteristics of receptors and ligand binding mechanisms. As a class B G-protein-coupled receptor, the dimerization/oligomerization of the N-terminal extracellular domain (NTD) of GLP-1R regulates ligand binding and signal transduction. Song et al. determined the dimerization affinity using SE-AUC, and the results were consistent with the results detected using NanoBiT technology. Combined with ligand binding experiments, the correlation between receptor dimerization and the negative cooperativity of ligand binding was clarified, providing biophysical evidence to interpret the interaction mechanism between GLP-1-class peptides and their receptors [66].

During the research on GLP-1-class peptide drugs, AUC runs through multiple links, including drug screening, formulation optimization, and stability evaluation. It can be used not only for an aggregation state analysis of peptide drugs themselves but also for research on peptide–receptor interactions and assembly mechanisms. However, we should also recognize that compared with other characterization methods, the sample throughput of AUC is moderate.

## 6. AUC Applications in EVs Research

Extracellular vesicles (EVs) are a general term for a bilayer membrane nanovesicle that is actively secreted by cells, including exosomes, microvesicles, and apoptotic vesicles. Exosomes with a diameter of 30–150 nm serve as important carriers that mediate intercellular material and information transmission. They show great clinical transformation potential in disease diagnosis and targeted drug delivery. EV samples are characterized by high heterogeneity and are often mixed with soluble proteins, lipoproteins, cell debris, and other impurities, making it difficult for a single characterization technology to achieve accurate analysis. During the evaluation and process optimization of EV isolation methods, Nix et al. adopted orthogonal verification via nanoparticle tracking analysis (NTA) and asymmetric flow field-flow fractionation coupled with multi-angle light scattering (AF4-MALS) to distinguish EV subpopulations from protein aggregates, providing a basis for evaluating the effectiveness of different isolation methods [69].

SV-AUC can be used for real-time monitoring of the sedimentation distribution of particles in a centrifugal field, and it can accurately distinguish vesicles from impurities combined with the A_260_/A_280_ absorbance ratio. Yu et al. systematically compared the performance of DLS, NTA, a NanoCoulter counter, and AUC in the characterization of milk- and urine-derived exosomes. They confirmed that AUC could clearly distinguish exosomes from casein, soluble proteins, and other impurities based on differences in the sedimentation behavior and nucleic acid-protein ratio. AUC could directly quantify the impurity removal efficiency of different purification strategies. Studies have shown that a single technology is insufficient for comprehensive exosome characterization. However, the combination of AUC with NTA, DLS, and other methods for orthogonal verification is expected to establish a more standardized and reproducible quality control system for exosomes [70].

## 7. AUC Applications in Other Recombinant Protein Products

Recombinant interferons are cytokines with antiviral, immunomodulatory, and anti-tumor activities whose biological functions are highly dependent on molecular structural integrity and stability. During formulation processes, interferons are prone to degradation or aggregation induced by stress factors such as temperature fluctuation and pH changes. They then form inactive fragments or high-molecular-weight aggregates. Without a stationary phase or labeling, AUC can directly resolve the sedimentation coefficient distribution of interferon monomers, degraded fragments, and aggregates. By fitting via the continuous c(s) distribution model, AUC quantifies the content of each component, providing key evidence for formulation screening and process parameter determination [71].

The recombinant human coagulation factor VIII is an essential biological product for the treatment of hemophilia A. Its purity and aggregation state directly affect product safety and efficacy. It has a large molecular weight and a complex structure, and it tends to form dimers, multimers, and other aggregates during production and storage. Conventional analytical methods, such as SEC, may exhibit deviation in the determination results due to column surface adsorption during analysis. By directly monitoring the sedimentation behavior in solution, AUC can effectively identify monomers, fragments, and aggregates of recombinant human coagulation factor VIII, serving as a reliable technical tool for product purity analyses. Studies have shown that SV-AUC can clearly resolve each component within a specific sedimentation coefficient distribution range and accurately calculate the monomer content and aggregate proportion [72].

Glycosylation modification of glycoproteins significantly affects molecular heterogeneity. Conventional methods, such as sodium dodecyl sulfate-polyacrylamide gel electrophoresis (SDS-PAGE) and SEC, have limited resolution and cannot distinguish glycoform variants from real aggregates. Based on the principle of synchronously capturing sample sedimentation signals via UV-visible and interference dual detection systems of AUC, Yu et al. established a novel glycoprotein analysis model in the independently developed AUC analysis software AUCAgent (v1.8.8). This enabled the direct calculation of the polysaccharide mass fraction in glycoproteins under native solution conditions. Using the ERBB-2 protein with 7 N-glycosylation sites as a model, the high stability and reproducibility of this method were verified. Another comparative AUC analysis method (glycoprotein analysis module of GUSSI 2.1.0 software) also exhibited extremely high stability, fully demonstrating the important application value of AUC in the glycan content analysis of glycoproteins [56].

## 8. Framework of AUC Methods for Biopharmaceuticals

Based on a synthesis of the aforementioned content, in this section, a universal standardized workflow for AUC method development and comprehensive validation applicable to the majority of biopharmaceuticals is presented. It is divided into three core components.

1. Method development strategy and condition optimization

The establishment of AUC detection methods requires the systematic optimization of experimental variables to guarantee satisfactory specificity. Key optimized factors are comprehensively sorted, including the rational selection of the SV-AUC or SE-AUC mode, rotor rotational speed, testing temperature, sample loading concentration, detection wavelength, extinction coefficient calibration, buffer density and viscosity correction, and the data fitting model configuration and data processing parameters. The matching principles of each parameter for different biopharmaceutical matrices are elaborated on in Table 1.

2. Full analytical method validation system

The core aim of analytical validation is to demonstrate that the developed AUC method is fit for its intended QC purpose, covering specificity, precision, accuracy, linearity, analytical range, sensitivity, and resolution. Furthermore, researchers may select corresponding validation items flexibly based on product characteristics and regulatory filing demands.

3. System suitability requirements for routine testing

System suitability criteria are defined as a critical guarantee to sustain stable and reliable analytical performance in each test run.

## 9. Summary and Prospects

As a first-principle-based classic biophysical analytical method, AUC provides high-resolution and high-precision detection data. It enables the real-time analysis of biological macromolecules in original formulation solutions under native conditions and accurately determines key physical properties such as intermolecular interactions. Compared with chromatographic methods that rely on stationary phases, AUC reduces the impact of column adsorption, sample dilution, and shear force on weak interaction systems. Compared with simple particle size characterization methods, AUC delivers more abundant information, including the sedimentation coefficient, distribution heterogeneity, molecular weight, and component proportion.

With the development of complex biological products such as bispecific antibodies, ADCs, AAV and LVV gene therapy vectors, VLP vaccines, mRNA-LNP vaccines, EV delivery systems, and glycoprotein products, QC has imposed higher requirements on analytical technologies. AUC exhibits unique advantages for the detection of such complex products. For the aggregate analysis of ADC drugs, it avoids quantitative deviation caused by filler adsorption or sample dilution in conventional SEC methods and provides more authentic aggregation state information. For the empty capsid ratio detection of AAV gene therapy products, it achieves high-resolution differentiation of empty capsids, partially packaged particles, and intact viral particles. For mRNA-LNP vaccines, AUC provides sedimentation/flotation profiles, buoyant density data, and nucleic acid optical signals to characterize particle size distribution of mRNA-LNPs; Moreover, the mRNA integrity and encapsulation efficiency inferred from these signals require orthogonal methods for definitive confirmation.

Notably, AUC has certain limitations, such as a moderate throughput, the need for expert data analysis, model dependence, possible optical artifacts, nonideality at high concentration, challenges with highly heterogeneous samples, and a lack of direct functional or infectivity readouts. Thus, it cannot replace all analytical technologies as a single platform. For complex biological products, reliable QC should be based on multi-technology orthogonal verification that combines AUC with SEC-MALS, AF4-MALS, CDMS, mass photometry, DLS, cryo-TEM, mass spectrometry, functional detection, and stability research. Looking ahead, with the standardization of AUC methodologies, automated data processing, the establishment of system suitability reference materials, and development of compliant software platforms, AUC is expected to play a more stable and standardized role in the quality by design (QbD) framework and the full life cycle quality management of biopharmaceuticals. A comprehensive comparison of the AUC assays for biopharmaceutical quality evaluation is shown in Table 2.

Moreover, a notable bottleneck limiting the standardized deployment of AUC across biopharmaceutical QC workflows should be recognized. That is, dedicated, unified reference materials exclusively designed for AUC system suitability assessment are currently insufficient, which raises notable barriers to completing method validation, as well as cross-laboratory data consistency comparison and regulatory filing. Based on industrial practice and official releases from the National Institutes for Food and Drug Control (NIFDC), two categories of commercially available reference substances widely adopted for routine AUC validation are summarized herein for reference: NIST mAb and NIST BSA standards, which serve universal system suitability controls during the AUC method establishment for antibody products, recombinant proteins, and peptide therapeutics; and the officially launched NIFDC AAV empty capsid system suitability reference material (Catalog No. 270041), which is commercially accessible and tailored to the AUC methodological verification of recombinant AAV gene therapy vectors.

## Figures and Tables

**Table 1:** Framework for analytical ultracentrifugation method construction for biopharmaceuticals.

Biopharmaceutical Product Category	Antibody Drugs		Viral Vector Products	VLP Vaccines		mRNA Vaccines	Conjugate Vaccines	Peptide Drugs	
AUC methods	SV	SE	SV	SV	SE	SV	SV	SV	SE
Rotor speed (rpm)	40,000–60,000	6000–9000–12,000	7000–20,000 or 45,000 (low temperature)	7000–40,000	14,000–17,000–20,000–40,000	10,000–30,000	25,000	50,000–60,000	20,000–31,000–40,000
Sample concentration	OD_280_ = 1.0 or 0.25–10 mg/mL	0.1–0.25–5–10 mg/mL	OD_260_ = 0.2–0.82 or capsid titer of 5.0 × 10^12^ vp/mL	OD_280_ = 0.7–1.0	OD_280_ = 0.18–0.35–0.7	OD_280_ = 0.3–0.8	OD_280_ = 0.3–0.5	20–80 μM or 0.14–2.0 mg/mL	2.2 μM–4.3 μM–6.5 Mm or 0.28–14 mg/mL
model selection	c(s) distribution or ls-g*(s)	monomer-“m-mer”-“n-mer” self-association model	c(s) distribution	c(s) distribution	Single ideal species model	c(s) distribution or ls-g*(s)	ls-g*(s)	c(s) distribution	monomer-“m-mer”-“n-mer” self-association model
Temperature	The experimental temperature is typically set at 20 °C or 25 °C, but sometimes, it is adjusted to 4–10 °C based on the stability of the sample and the rate of sedimentation. Temperature equilibration is reached in approximately 1–2 h								
Extinction coefficients	Extinction coefficients can be obtained through information such as amino acid sequences and nucleic acid sequences.								
Buffer density and viscosity	Buffer density and viscosity can be calculated using the program SEDNTERP or measured using densitometer and viscometer.								

**Table 2:** Comprehensive comparison of analytical ultracentrifugation assays for biopharmaceutical quality evaluation.

Biopharmaceutical Product Category	Antibody Drugs	Viral Vector Products	Vaccines	Peptide Drugs	Extracellular Vesicle
AUC methods	SV and SE	SV	SV	SV and SE	SV
Quality attributes	Molecular size variants; self-association; antigen–antibody complexes	Empty/full capsid ratios; distinguishing empty capsid particles, intermediate particles, and intact viral particles	Viral particle assembly; LNP formulation characterization; polysaccharide proportion in vaccines	Oligomer distribution; interaction between receptor and peptide; aggregation constants	Distinguishing vesicles from impurities
Orthogonal methods	SEC; SEC-MALS; CE-SDS; ITC; MALDI	MP; CDMS; SEC-MALS	TEM, DLS, MALS	SEC, TEM, CD	NTA; DLS; AF4-MALS
Key advantages	Original formulation solutions under native conditions; real-time analysis; high-resolution; multiple quality attributes				
Limitations	Moderate throughput; model dependence; nonideality at high concentration; challenges with highly heterogeneous samples; lack of direct functional or infectivity readouts				
Regulatory relevance	AUC is used as an orthogonal analytical method in comparative analytical assessments of therapeutic protein biosimilars by the FDA. AUC is recognized and validated as an analytical approach to quantify AAV empty, partial, and full capsids, and it is accepted by the FDA, NMPA, and EMA.				

## Data Availability

No new data were created or analyzed in this study. Data sharing is not applicable to this article.
